# Recent developments in isolating methods for exosomes

**DOI:** 10.3389/fbioe.2022.1100892

**Published:** 2023-01-13

**Authors:** Jiahui Gao, Ang Li, Jie Hu, Linxiang Feng, Liu Liu, Zuojun Shen

**Affiliations:** Department of Clinical Laboratory, The First Affiliated Hospital of USTC, Division of Life Sciences and Medicine, University of Science and Technology of China, Hefei, Anhui, China

**Keywords:** exosomes, extracellular vesicles, isolation methods, isolation technologies, review

## Abstract

Exosomes are the smallest extracellular vesicles that can be released by practically all cell types, and range in size from 30 nm to 150 nm. As the major marker of liquid biopsies, exosomes have great potential for disease diagnosis, therapy, and prognosis. However, their inherent heterogeneity, the complexity of biological fluids, and the presence of nanoscale contaminants make the isolation of exosomes a great challenge. Traditional isolation methods of exosomes are cumbersome and challenging with complex and time-consuming operations. In recent years, the emergence of microfluidic chips, nanolithography, electro-deposition, and other technologies has promoted the combination and innovation of the isolation methods. The application of these methods has brought very considerable benefits to the isolation of exosomes such as ultra-fast, portable integration, and low loss. There are significant functional improvements in isolation yield, isolation purity, and clinical applications. In this review, a series of methods for the isolation of exosomes are summarized, with emphasis on the emerging methods, and in-depth comparison and analysis of each method are provided, including their principles, merits, and demerits.

## 1 Introduction

Exosomes are membranous vesicles released by intracellular multivesicular bodies that can be involved in intercellular communication, angiogenesis (Geng et al., 2020), and tumor formation (Li et al., 2021). They play an important role in the early diagnosis of cancer (Wu et al., 2021), infectious diseases (Shrivastava et al., 2021), and cardiovascular diseases (Neves et al., 2022). Although biological properties and functional characteristics of exosomes have been intensively studied, the high heterogeneity of exosomes in terms of size, cargo, and origin (Han et al., 2021a), as well as the presence of nanoscale contaminants such as lipoproteins, retroviruses, etc., have limited the development of methods for isolation and purification of exosomes.

So far, the methods for exosome isolation are constantly improving. The traditional methods mainly include differential ultracentrifugation, size-based isolation, and polymer-based precipitation. These methods can be used to handle large volume samples or to obtain high purity samples. However, high yield and purity cannot be guaranteed by most exosome isolation methods. Some isolation methods are not only tedious and costly, but also require specialized instruments. Like ultracentrifugation, exosomes are easily damaged mechanically under the action of centrifugal force, and it is difficult to effectively maintain the bioactivity and morphological integrity of exosomes.

**FIGURE 1:**
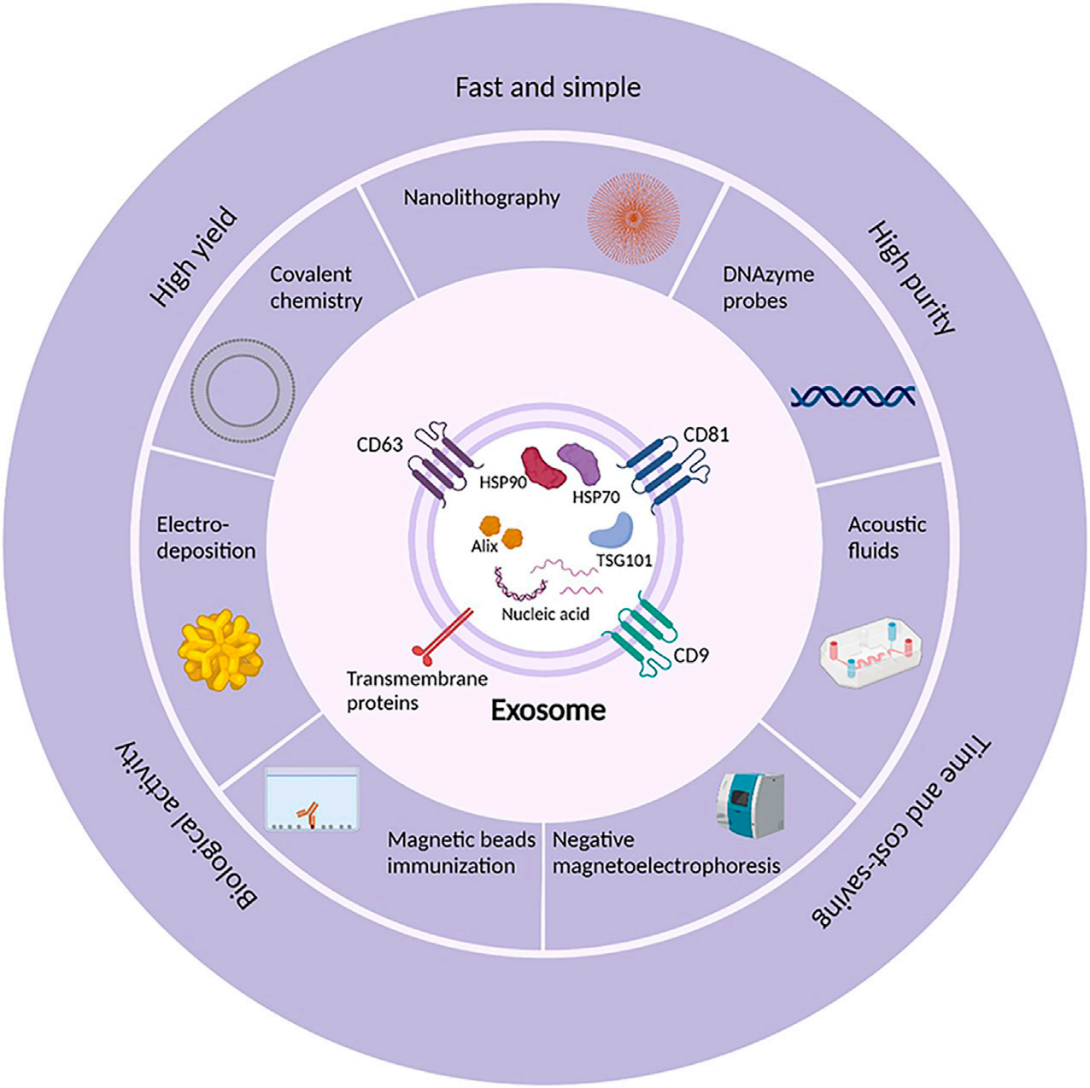
The emerging isolation methods and technologies and the hallmarkers of exosomes. Exosomes are usually found to express markers like CD63, CD81, CD9, HSP70, HSP90, TSG101, and Alix. Currently, exosomes can be isolated by a variety of methods and technologies such as nanolithography, electro-deposition, immunomagnetic beads, covalent chemistry, DNAzyme probes, and negative magneticelectrophoresis to achieve the isolation efficiency of simple and rapid, high yield and purity, time and cost-saving.

In recent years, emerging methods and technologies, such as microfluidics (Hassanpour Tamrin et al., 2021), nanolithography, electro-deposition, immunomagnetic beads, and covalent chemistry, have significant effects on isolation of exosomes (Figure 1). As an example, field flow isolation (Kim et al., 2022a), label-free magnetic isolation (Zeng et al., 2021), and functional micro/nanostructures (Lim et al., 2022) can be used to extract exosomes efficiently. Meanwhile, microfluidic devices have been developed to integrate the isolation and analysis of exosomes into a platform where exosomes can be directly characterized and analyzed for downstream genomics and proteomics. In addition, the extracellular vesicles on demand (EVOD) chip can be used to isolate and analyze lung cancer derived exosomes (Kang et al., 2020).

While ensuring high yield and purity, the bioactivity and structural integrity of exosomes can be maintained. Only on the basis of good separation products can exosomes be effectively studied. Therefore, the selection of appropriate separation technique is the prerequisite of exosome research. In this review, the improvements and innovations on the traditional methods, such as dichotomic size-exclusion chromatography (Guo et al., 2021a), ultracentrifugation in combination with size-exclusion chromatography (Vergauwen et al., 2021) are introduced. The emerging methods proposed in the last two years are elaborated, such as label-free separation of nanoparticles based on the principle of particle ferrohydrodynamics (Liu et al., 2020) and separation by DSPE-PEG derivatives in combined with biotin-avidin systems (Wei et al., 2021).

## 2 Improvements of traditional methods for exosome isolation

Traditional methods for exosome isolation include ultracentrifugation-based methods, size-based methods (size-exclusion chromatography and ultrafiltration), precipitation, and immunoaffinity capture. The first three methods are mainly based on the size and density of exosomes, and the last method is mainly based on the specific binding between antibodies or aptamers and exosomal signature proteins. The comparison of each method is summarized in Table 1. On this basis, many researchers have integrated and innovated on the existing methods. For example, size-exclusion chromatography combined with iodixanol density gradient centrifugation was used for isolation, aiming to achieve high yield and purity (Alameldin et al., 2021).

**TABLE 1:** Comparison of traditional isolation methods of exosomes.

Method	Principles	Purity	Recovery	Samples	Application scenarios	Advantages	Disadvantages	References
Differential ultracentrifugation	Size and density	Medium	Low	Blood, urine, saliva, cerebrospinal fluid, cell culture medium, tissues (such as Melanoma, Alzheimer’s disease)	Suitable for the functional study of exosomes	Relatively high purity	Time-consuming	Ter-Ovanesyan et al. (2021); Correll et al. (2022)
						Widely used	Exosome damage	
						Large sample capacity	Large sample volumes	
							Expensive instrumentation	
Density gradient centrifugation	Size and density	High	Low	Cell, cell subcomponent, nucleic acid, virus, protein complex	The method is classical and suitable for omics research	High purity	Time-consuming	Langevin et al. (2019)
				Exosomes can be isolated from non-vesicle particles		High recovery rate	Exosome damage	
							Large sample volumes	
Size-exclusion chromatography	Size	High	Relatively low	Blood, urine, cell culture medium	It is suitable for most exosome research scenarios, especially those requiring high purity, omics, and large volume samples	Cost-saving	Specialized instrumentation	Guo et al. (2021a)
				It is widely used for the separation of exosomes from blood and urine. For plasma		Time-saving	Nanoscale contaminants (e.g., lipoproteins)	
				SEC + UC can obtain higher purity than UC, precipitation and microfluidic		Fast and simple		
						Maintain the biological activity and integrity of exosomes		
Ultrafiltration	Size	Low	High	Blood, urine, cell culture medium	It is often used in combination with other methods and is suitable for the study of sample concentration	Simple	Low purity	Han et al. (2021b); Kim et al. (2021)
						Time-saving	Clogging membrane pores	
						Relatively cheap		
						High yield and efficiency		
Precipitation	Solubility	Low	Relatively high	Blood, urine, cell culture medium	Suitable for studies with very low purity requirements that do not require omics studies	High yield	Co-isolation of non-EV particles (e.g., lipoproteins)	Kamei et al. (2021)
						Widely used	Polymers may affect downstream analysis	
						Relatively cheap		
						Commercialized kits		
Immunoaffinity capture	Specific binding	High	Relatively low	Blood, urine, cell culture medium	Suitable for the research that requires the separation of specific exosome subgroups and high purity	High purity	Expensive	Mondal and Whiteside, (2021)
						Subpopulations can be isolated	Low yield	
						Can be integrated with detection systems	Nanoscale contaminants	
							Intermolecular cross-reactions	

Differential ultracentrifugation is considered to be the “gold standard” method for exosome isolation, which can effectively isolate microscopic particles such as bacteria, organelles, and exosomes (Ter-Ovanesyan et al., 2021). To minimize the effect of Tamm-Horsfall protein polymer on the yield and purity of urinary extracellular vesicles, differential ultracentrifugation combines Tamm-Horsfall protein polymer reduction and alkaline washing to reduce common protein contaminations (Correll et al., 2022). Density gradient centrifugation (Langevin et al., 2019) is a time-consuming method due to the high-density medium (sucrose or iodixanol, etc.) is added to the layer which can be used to isolate extracellular components (such as exosomes, apoptotic vesicles, and protein aggregates). One-step sucrose cushion ultracentrifugation can be used to achieve the extraction of MSCs-derived exosomes (Gupta et al., 2018). Li et al. (2018) and Luo et al. (2021) have improved this method to maximize the recovery and purity of exosomes while minimizing the exosome damage. In contrast to sucrose-based gradient centrifugation, the isolation of subpopulations of extracellular vesicles can be achieved by high-resolution iodixanol density gradient centrifugation more efficiently (D'Acunzo et al., 2022).

**FIGURE 2:**
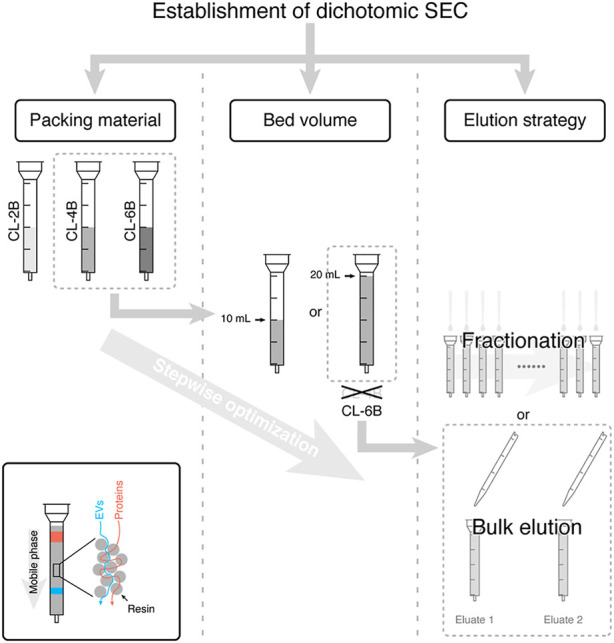
Establishment of dichotomic SEC. The dichotomic SEC separation with 2 bulk elution steps was sufficient for the isolation of the extracellular vesicle. When the packing material is CL-6B and the bed volume is 20 mL, it will be the best separation performance. Reproduced with permission from (Guo et al., 2021a).

To improve the isolation purity, researchers have tried to combine ultracentrifugation and ultrafiltration with size-exclusion chromatography for the isolation of exosomes (Guan et al., 2020; Shu et al., 2021; Turner et al., 2022). Studies have shown that ultrafiltration combined with size-exclusion chromatography can reduce the level of impurity cytokine (interleukin-10) in isolated exosomes. The combination of various methods can effectively reduce the presence of nanoscale contaminants, improve the separation purity, while maintaining the natural properties of exosomes. In addition to methods combination, other researchers have proposed dichotomic size-exclusion chromatography for exosome isolation, and found that CL-6B 20 mL column had the best performance with higher isolation yield and tighter EVs peaks (Figure 2) (Guo et al., 2021a). Nowadays, qEV size-exclusion columns exist on the market. Highly active exosomes can be extracted from a wide range of samples within 15 min based on the principle of molecular particle size segmentation (Cardoso et al., 2021). Besides, studies have shown that tangential flow filtration is more suitable for ultrafiltration processes than traditional dead-end filtration (Han et al., 2021b; Kim et al., 2021). In tangential flow filtration, the potential clogging can be effectively reduced, and thorough filtration under the effect of parallel flow dynamics can be ensured. Precipitation-based and immunoaffinity-based kits have been widely used for exosome isolation (Kamei et al., 2021; Mondal and Whiteside, 2021). Furthermore, polysaccharide chitosan could facilitate the isolation of small extracellular vesicles (Kumar et al., 2021). And researchers (Crescitelli et al., 2021) have summarized the process of isolating and characterizing subpopulations of EVs from tissues.

## 3 Emerging methods for exosome isolation

Although traditional isolation methods of exosomes such as ultracentrifugation and kit extraction are widely used for scientific research, these methods have certain limitations such as low isolation yield and purity, time-consuming. The practice of exosomes in clinical applications (such as point-of-care testing, POCT) is seriously hindered. In recent years, with the development of microfluidic chips (Lu et al., 2022), it has become possible to handle or manipulate small volumes of liquid in microplates and microchannels. Microfluidic devices are used to achieve the integration of isolation and identification on a microchip (Ullah Khan et al., 2021). In addition, the multidirectional reference of the development of exosome isolation techniques can be provided by applications of nanomaterials, immunomagnetic beads, and covalent chemistry. They play an important role in improving the isolation yield and purity and maintaining the bioactivity of exosomes. The principles, advantages, and disadvantages of each method are summarized in Table 2.

**TABLE 2:** Comparison of emerging isolation methods of exosomes.

Method	Principles	Purity	Recovery	Advantages	Disadvantages	References
Asymmetric Flow Field-Flow Fractionation	Size	High	Relatively high	Label-free	Capacity limitation	Manning et al. (2021)
				Little damage to exosomes	Required specialized equipment	
				Subpopulations can be isolated	Co-isolation with non-EVs particles	
Deterministic Lateral Displacement	Size	Low	High	Label-free	Clogging membrane pores	Smith et al. (2018); Hochstetter et al. (2020)
				Time-saving	Specific instrumentation	
				Labor-saving	Co-isolation with non-EVs particles	
				Maintain the biological activity of exosomes		
Dielectrophoretic	Size	Relatively low	Relatively low	Label-free	Low purity	Tayebi et al. (2021); Zhang et al. (2022a)
				High selectivity	The device will overheat	
				High controllability	Tumor-derived exosomes are not separable	
				Little damage to exosomes		
Acoustic Fractionation	Size	High	Relatively high	Simple	Specialized instrumentation	Wang et al. (2021)
				Label-free	Co-isolation with non-EVs particles	
				Good biocompatibility		
Non-contact microfluidics	Viscoelastic media flow	Relatively high	Relatively high	Less contaminations	Relatively low sensitivity	Rodriguez-Quijada and Dahl, (2021)
					Weak anti-interference ability	
EXODUS	Size	High	Relatively high	Fast	Capacity limitation	Chen et al. (2021)
	Specific binding			No clogging	Required expertise and specialized equipment	
				Repeatability		
				Isolation and detection integration		
Exo-CMDS	Charge	High	Relatively high	Fast	Membrane clogging	Zhao et al. (2022)
				Low cost	Relatively expensive	
				High purity	Co-isolation with non-EVs particles	
				High selectivity		
3D ZnO Nanoarrays	Acoustic fluid	Relatively low	Relatively low	Fast	Specialized	Hao et al. (2020)
				High sensitivity	Relatively expensive	
				Multifunction		
				Downstream analysis is possible		
Lipid microarrays	Specific binding	High	Relatively low	Fast	Expensive	Liu et al. (2021)
				High sensitivity	Low yield	
				Small volume samples	Difficult to apply to the clinic	
				Inherent antifouling properties		
Capture by immunomagnetic beads	Size	High	Relatively low	High purity	Capacity limitation	Cheng et al. (2022); Zhang et al. (2022c); Zheng et al. (2022)
	Specific binding			Maintain the biological activity and morphological integrity of exosomes Easy to combine with specific analysis tools	Chelator adverse effects	
					Expensive	
					Membrane clogging	
					Co-isolation with non-EVs particles	
Synthetic polypeptide	Specific binding	Relatively high	Relatively low	Clinical application	Expensive	Bathini et al. (2021)
Vn96 captures exosomes				High-throughput analysis	Low yield	
				Maintain the biological activity and morphological integrity of exosomes	Relatively troublesome	
ExoSD	Size	Relatively high	Relatively high	Relatively high purity	Specialized	Yu et al. (2021)
				Multifunction	Capacity limitation	
				Relatively high recovery rate		
Capturing exosomes with covalent chemistry	Covalent chemistry	Relatively high	Relatively low	Fast and automatic	Relatively complex	Dong et al. (2020); Han et al. (2020); Liu et al. (2022b); Sun et al. (2022)
				High efficiency	Relatively expensive	
				Maintain the integrity of exosomes	Relatively specialized	
				Easy to combine with specific analysis tools	Not suitable for larger volume samples	
					Co-isolation with non-EVs particles	
					Downstream analysis may be affected	

### 3.1 Integrated microfluidic

The microfluidic technology refers to the manipulation and processing of tiny fluids in micropores. The isolation and characterization of exosomes in a single step process are allowed, with microfabrication technologies to develop chip-based microfluidic systems. The comprehensive microfluidic devices based on immunoaffinity capture (Mondal and Whiteside, 2021), size-exclusion chromatography (μSEC) (Leong et al., 2022), and ultracentrifugation (Kang et al., 2021) have been developed for the efficient isolation of exosomes. The rapid isolation of exosomes from minute volumes of biofluids, real-time characterization, and *in situ* diagnosis of exosomes can be realized by the combination of microfluidic devices and signal detection platforms. It is important for the non-invasive disease detection. The advantages of microfluidics are convenience, high efficiency, ease of automation, integration, and portability. However, the sample throughput is low because the sample is fed in the fingertip volume. The microfluidic devices can also be combined with exponential isothermal amplification (EXPAR) of miRNAs (Qian et al., 2022) for the isolation and identification of exosomes. Meanwhile, integrated microfluidic platforms consisting of a single-cell capture chip and a spatially coded antibody barcode chip can also be used to explore single-cell heterogeneity (Song et al., 2022).

#### 3.1.1 Asymmetric flow field-flow fractionation

Field flow fractionation can be used to isolate macromolecules (Manning et al., 2021). The molecules passing through a flat channel are subjected to both horizontal and vertical flow fields. When the flat channel is closed above and only open below is called Asymmetric Flow Field-Flow Fractionation (AF4). AF4 is mainly based on the size to isolate nanoscale soluble particles (Wu et al., 2020). There is a high correlation between the particle size and diffusion coefficient (Zhang and Lyden, 2019). The isolation of particles can be affected by diffusion coefficient, so that the key of the isolation of AF4 is the particle size. The bottom of AF4 is a semi-permeable membrane with the specific size. The molecules with sizes smaller than the cut-off size can pass through the semi-permeable membrane, and components larger than the cut-off size are retained. Studies have shown that AF4 can identify particles from the nanometer to the micrometer scale, and has a large dynamic range and high resolution, with a resolution of up to 1 nm (Marioli et al., 2019).

Compared with other isolation methods, AF4 is no labeling, no magnetic beads, and has a high reproducibility. However, AF4 can only be used to process small volume samples and cannot be used to screen particles with same size and different morphology. The complicated production process is a stumbling block to clinical applications (Berger et al., 2021). Furthermore, asymmetric depth-filtration (DF) can isolate extracellular vesicles by immobilizing extracellular vesicles on the surface and within the depth of porous medium, then recovering them by reversing the carrier flow through the filter. Compared to the optimized three-step isolation procedure, DF is more suitable for the separation of plasma extracellular vesicles (Chernyshev et al., 2022).

#### 3.1.2 Deterministic lateral displacement

Deterministic Lateral Displacement (DLD) is particle size-based isolation, and can be used for the isolation of exosomes (Hochstetter et al., 2020). The system consists of an ordered array of microcolumns, where the gaps and offsets between microcolumns determine the diameter of particles that can pass through. The cut-off size parameter between the serrated and displaced modes in DLD is called DLD critical diameter (Dc) and is mainly determined by the column shape, the column gap, and the array gradient (Ho et al., 2020). Biofluids flow through the array. When the particle size is smaller than the critical size, particles will follow the initial streamline through the array gap. When the particle size is larger than the critical size, particles are laterally displaced by the column impact in a cross-order flow line. Thanks to the continuous flow column array gradient, DLD is widely used for the isolation of bacteria, viruses, and yeast.

However, due to the fluid dynamics involved at the micron or even nanoscale, it is more difficult in fabrication and sorting. Smith et al. (2018) refined on a nanoDLD chip with an elevated fluid flow rate of ∼900 μL/h compared to conventional arrays. As early as 2016, Zeming KK et al. designed a DLD device for the isolation of nanoparticles, mainly by varying the concentration of salt ions to modulate the interaction between nanostructures and particles. To a certain extent, the bioactivity and morphological integrity of exosomes can be maintained. In addition, exosomes can be isolated by DLD with high separation yield and low consumption, and without labeling. However, the chip is more prone to clogging than conventional isolation methods, resulting in lower isolation purity.

#### 3.1.3 Dielectrophoretic isolation

Dielectrophoretic isolation (DEP) is based on the translational motion of neutral particles subjected to dielectric polarization in a non-uniform electric field, including positive and negative dielectrophoresis. Smaller particles enter the DEP high field region around the edges of microelectrodes, and larger particles enter the DEP low field region between electrodes. In an inhomogeneous symmetric electric field, particles are subjected to different local electric field strengths on both sides, generating an electrostatic force, i.e., the dielectric electrophoretic force. The dielectric electrophoretic force is determined by the size of suspended particles, dielectric constant, and electric field strength. Since the suspended particles and the medium have different dielectric constants, particles will move in the direction of a stronger or weaker electric field. Compared with other separation methods, DEP is more selective and controllable for the rapid separation of exosomes, and no labeling (Tayebi et al., 2021; Zhang et al., 2022a).

To extend the isolation efficacy of DEP, Ayala-Mar et al. (2019) proposed a DC insulator-based dielectrophoresis (DC-iDEP). iDEP is a variant of DEP that can isolate particles based on the size. Since the insertion of electrodes in the chip is not required, the fabrication process is greatly simplified (Shi et al., 2018). Researchers designed a microdevice consisting of two electrically insulated column fraction channels, and the isolation efficiency of exosomes can be assessed by analyzing the particle size of the recovered fraction in DC-iDEP. Although iDEP can capture exosomes efficiently and differentiate subpopulations of exosomes to a certain extent, it is undeniable that the isolation purity is low and the device is subject to overheating. Zhao et al. (2021) proposed an ExoDEP-chip microfluidic device which contains a large number of microwells in the DEP chamber with forked-finger DEP electrodes, and single polystyrene (PS) microsphere is immobilized in the micropores. Exosome capture is achieved by pre-conjugated antibodies on the PS surface. ExoDEP-chip is more flexible and less complex in terms of the equipment and operation.

Additionally, a label-free magnetic isolation system that can be used to extract small extracellular vesicles (sEVs) from cell culture supernatants based on negative magnetoelectrophoresis was presented (Zeng et al., 2022). The strong magnetic field within the microchannel is generated by Four NdFeB magnets (N52). Particles are concentrated in the isolation channel by sheath flow and are repelled by the magnetic pole array into the center of isolation channel by the strong magnetic force, with particles of different sizes separated in different exits. Higher recovery and purity can be achieved by using biocompatible ferromagnetic fluids with recovery up to 85.80% and separation purity up to 80.45% compared with other isolation methods. Since the isolation of sEVs by negative magnetoelectrophoresis is performed in the magnetic solution, which is likely to affect the bioactivity of sEVs, so the magnetic solution is highly required.

#### 3.1.4 Acoustic fractionation

**FIGURE 3:**
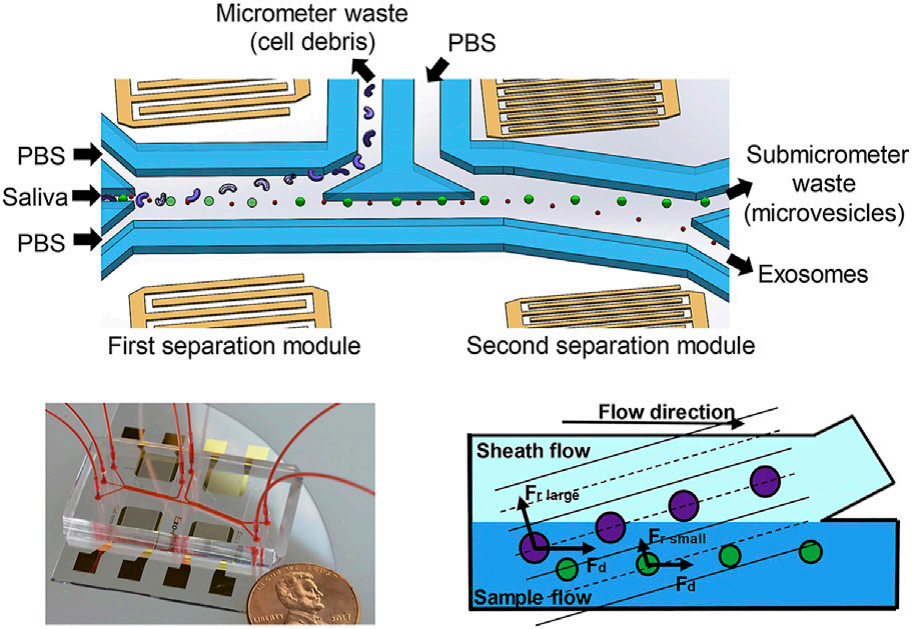
Schematic diagram of an acoustic fluid device for salivary exosome isolation. Micrometer and submicrometer waste can be separated by using 20 MHz and 40 MHz surface sound waves. During the separation process, the particles are subjected to the acoustic radiation force and the drag force. Exosomes can be separated in the second separation module. Reproduced with permission from (Wang et al., 2020).

Acoustic Fractionation can isolate particles based on the size and density (Wang et al., 2021). Particles are subjected to acoustic standing waves (SAW) in the micro channel. Acoustic radiation force and Stokes resistance can be generated. For larger particles, the acoustic radiation force dominates, and for smaller particles, the Stokes resistance dominates. Acoustic fractionation is no labeling and simple to operate. Relevant studies have shown that exosomes can be separated from biofluids with a purity of up to 98% and a separation rate of up to 82%. Wu et al. (2017) designed an acoustic flow control platform integrated onto a single chip that can isolate exosomes directly from blood samples. The salivary exosomes can be directly isolated by the device (Figure 3) (Wang et al., 2020). However, its clinical application is limited by the need for specialized instrumentation. With the development of single vesicle technique, researchers proposed that single vesicle technique combined with atomic force microscopy (AFM) and ultrasensitive TiN-NH-localized surface plasmon resonance (LSPR) biosensors could be used to characterize and quantify exosome-associated proteins for liquid biopsy *in vivo* (Silva et al., 2021; Thakur et al., 2021).

In addition to acoustic fractionation, a FerroChip device combining ferrohydrodynamic and microfluidic systems has been proposed to isolate extracellular vesicles (Liu et al., 2020). Under the action of ferromagnetic fluid sheath flow, premixes of antimagnetic nanoparticles and extracellular vesicles with ferromagnetic fluid enter straight microchannels. Small particles are allowed to migrate at a slower rate by difference of the particle size, while extracellular vesicles migrating at a faster rate, resulting in spatial isolation at the channel exit. The microfluidic chip is no labeling, and extracellular vesicles will be captured by a continuous flow and size-dependent manner. It has been reported to achieve 94.3% recovery and 87.9% isolation purity.

#### 3.1.5 Non-contact microfluidics

The separation principle of non-contact microfluidics is that the fluid containing the electric vehicle encounters a sheath flow along the wall of the microchannel in a microfluidic system based on viscoelastic media flow (Rodriguez-Quijada and Dahl, 2021). After applying the elastic lifting force generated by the viscoelasticity of fluids, exosomes and other extracellular components are driven towards the centerline of the microchannel according to size. Larger particles eventually reach the centerline, thus achieving the isolation of exosomes. Besides, researchers have pointed out that extracellular vesicles can be rapidly enriched and detected by phospholipids and transmembrane proteins in microfluidic chips (Ren et al., 2022). Li et al. (2022) proposed a 3D-SiO_2_ porous chip, which applied to prostate cancer (PCa) staging in mice and early detection of clinical PCa patients. Results show that early detection rate of clinical patients can be improved by non-contact microfluidics. Combining nanoscale porous properties with exosome-specific markers, the sensitivity of biosensing can be improved by the 3D-SiO_2_ porous chip greatly.

#### 3.1.6 Other approaches

##### 3.1.6.1 Capture by ultra-fast exosome isolation system

**FIGURE 4:**
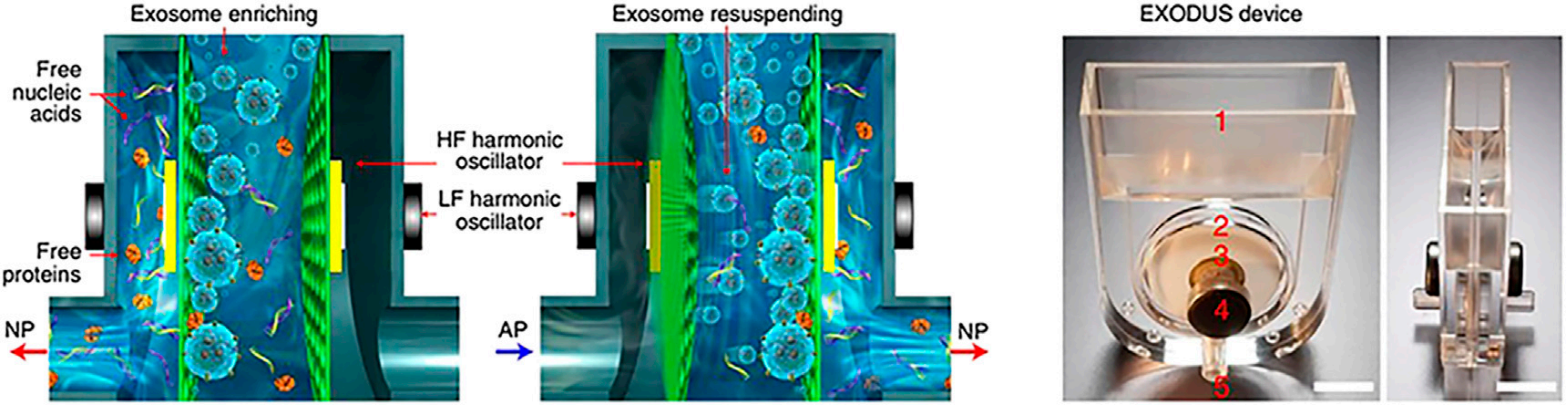
The mechanism of NPO and the schematic diagram of the EXODUS device. Exosomes can be isolated ultra-rapidly using negative pressure oscillations and double-coupled harmonic oscillator membrane vibrations. Reproduced with permission from (Chen et al., 2021).

Chen et al. (2021) proposed an ultra-fast exosome isolation system (EXODUS) for the efficient isolation and detection of exosomes. The automatic purification of exosomes from different biofluids is allowed by EXODUS. The key part is the isolation of exosomes by membrane vibrations generated by negative pressure oscillations and dual coupled resonators. Dual frequency transverse waves on the membrane are generated. Nanoporous membranes and cartridge oscillations are used to suppress membrane fouling effects to achieve clog-free and ultra-fast purification of exosomes. There is a dual-filter cuvette with one left and right outlet in the system, both of which are connected to nanoporous anodic aluminum oxide membranes (Patel et al., 2020). By switching the direction of negative pressure and air pressure, periodic negative pressure oscillations are generated across anodic aluminum oxide membranes. Periodic switching of negative pressure is used to allow the passage of small particles and fluids while exosomes are retained in the central chamber. With high-frequency harmonic oscillations generated by the nanopore disk resonator and low-frequency harmonic oscillations used throughout the device, dual-frequency transverse waves and acoustic fluid resuspend surface particles, effectively suppressing particle aggregation and scaling effects (Figure 4). They took 113 clinical urine samples for the functional validation of EXODUS.

Compared to the conventional isolation methods such as precipitation and ultracentrifugation, EXODUS has advantages in terms of isolation speed, yield, and purity. Moreover, the processing time of samples is short, narrowing down to 10 min for a 10 mL of urine. The purity of exosomes was verified by the uromodulin (Droste et al., 2021). In comparison with precipitation and membrane affinity, the band intensity of uromodulin was lower, indicating the good purification performance of EXODUS. Purification of 15 mL cell culture medium with EXODUS resulted in removal of approximately 99% of protein impurities and recovery of approximately 90% of exosomes. iTEARS can rapidly isolate exosomes from small volumes of tears (∼10 µL) by using EXODUS (Hu et al., 2022). This system has important implications for the establishment of the precision medicine based on tear exosomes.

##### 3.1.6.2 Capture by the centrifugal microfluidic disc system

The centrifugal microfluidic disc system (Exo-CMDS) is proposed by Zhao et al. (2022) for the isolation of exosomes. The automated centrifugal microfluidic disc system is combined with a functionalized membrane to form Exo-CMDS. The centrifugal microfluidic disc contains two functional membranes, one of which is used to filter blood cells and larger particles, and the other is used to enrich exosomes. The membrane is a quaternized regenerated cellulose membrane with a positive charge that adsorbs the negative charge from the exosome surface. In this system, exosomes can be adsorbed onto the membrane by the loading buffer (ExoL), and exosomes on the membrane can be eluted by the separation buffer (ExoI). The advantages of Exo-CMDS over conventional isolation methods are higher isolation yield and purity. In addition, Exo-CMDS has higher sensitivity, higher specificity, and low cost. It took only 8 min to process blood samples with a volume of less than 300 μL. At the same time, the novel aptamer fluorescence system (Exo-AFS) can be incorporated with Exo-CMDS to detect exosomal surface proteins, which is useful for diagnosing cancer. Furthermore, the quantification of specific subpopulations is allowed by the droplet-based extracellular vesicle analysis (DEVA) (Yang et al., 2022a). In this platform, droplet generation, processing, and analysis can be performed simultaneously, enabling high-throughput digital determination of extracellular vesicles.

##### 3.1.6.3 Capture by 3D ZnO nanoarrays

Hao et al. (2020) presented a multifunctional 3D nanostructured array by using the acoustofluidic technology. The acoustic sensor was integrated with an acoustofluidic reactor to build a 3D ZnO nanoarray within a capillary microchannel, which can capture nanoscale biomolecules. The ZnO nanoarray has a wide elastic range in length, density, and diameter. At the same time, Ag nanoparticles were deposited onto the ZnO nanoarrays to obtain ZnO-Ag capillary elements for the detection of exosomes, DNA, oligonucleotides. The functional validation of ZnO-Ag capillaries was performed by the finite-difference time-domain (FDTD) simulations. The ideas of the development of microanalytical devices can be provided by the enhanced optical sensing of ZnO-Ag. Zhang et al. (2019) developed an integrated platform for the analysis of exosomes (ExoProfile chip). The 3D porous serpentine nanostructures were innovatively constructed, and multiplexed detection of exosomes was allowed by this chip. In addition, it has shown that the dual-selective fluorescent nanosensor (Feng et al., 2022), 3D nanomachine based on Janus wireframe DNA cube (Xu et al., 2022), and 3D molecular beacon based on cubic DNA nanospaces (Mao et al., 2022) can be used for the characterization and analysis of exosomes (Guo et al., 2021b).

### 3.2 Immunocapture on a chip

The immunological methods for exosome isolation are mainly to capture the characteristic proteins on the surface of exosomes (such as CD63, CD9, and heat shock proteins) by the modified antibodies or aptamers. The biotinylated antibodies or aptamers and immunomagnetic beads can be used for exosome isolation. The functionalized magnetic beads have been widely used for the exosome isolation by combining microfluidic devices with immunological methods. Meanwhile, the integration of the exosome isolation and detection can be realized by the combination of methods. For example, Zhang et al. (2022b) performed label-free detection of PD-L1 in exosomes based on the surface plasmon resonance biochip (SPR-ExoPD-L1) to provide effective information for the diagnosis and treatment of cancer. Either a fluorescent biosensor (Chen et al., 2022) or a ROX-labeled aptamer (ROX-Apt) assembled on the surface of MoS2-AuNSs specifically bound to CD63 can be used to detect exosomes (Pan et al., 2022).

#### 3.2.1 Lipid microarrays

**FIGURE 5:**
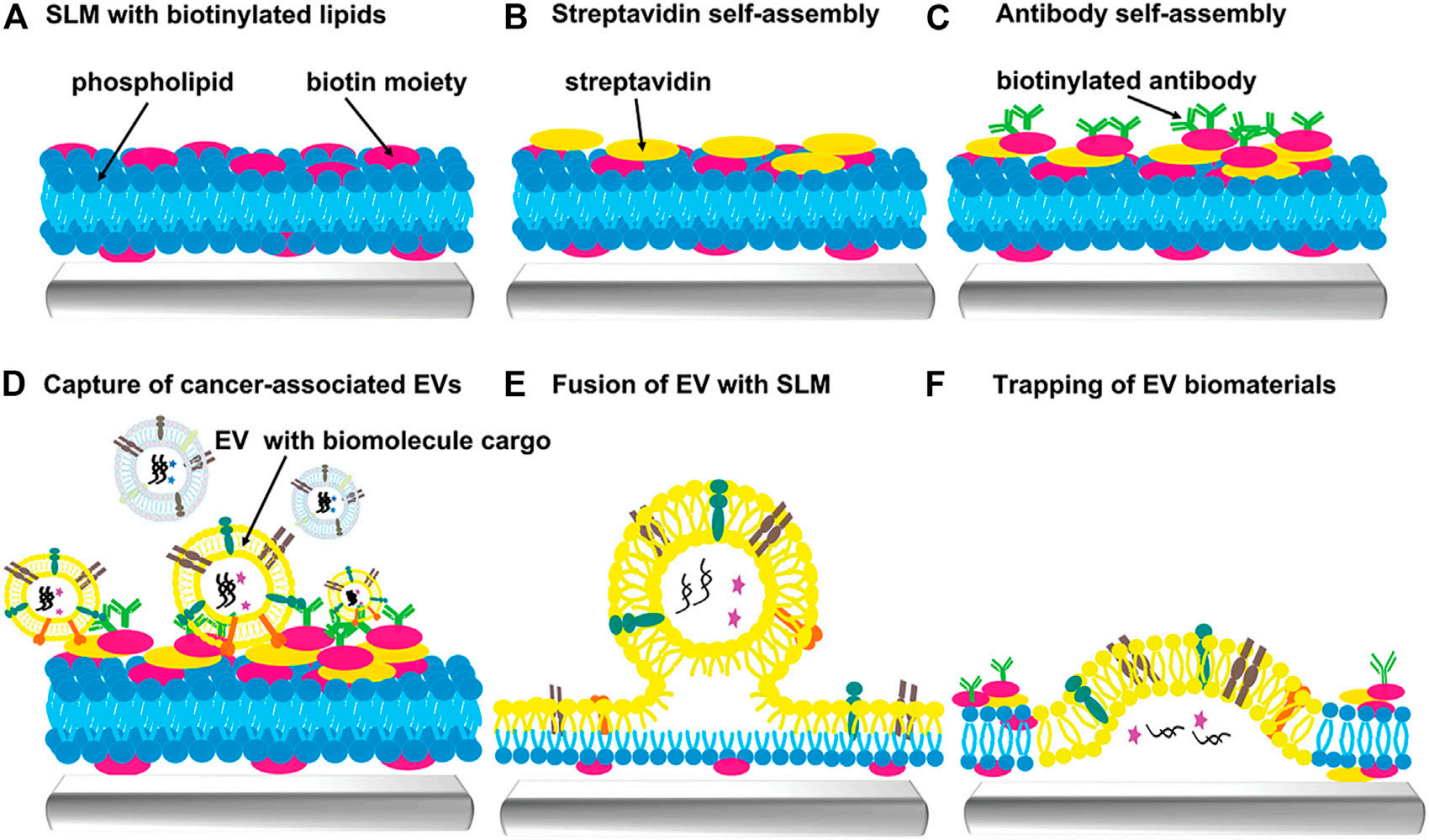
Scheme of extracellular vesicles capture by supporting lipid membranes (SLM). **(A)** Fabrication of biotinylated SLM arrays can be achieved by L-DPN. **(B)** Biotinylated SLM arrays are encapsulated with streptavidin. **(C)** Biotinylated antibodies can bind to the SLM arrays to capture specific EVs. **(D)** Cancer-associated EVs are captured on the SLM arrays. **(E)** Fusion of EV with SLM. **(F)** Trapping of EV biomaterials (such as RNA, proteins). Reproduced with permission from (Liu et al., 2021).

Lipid microarray is a novel strategy for exosome capture. Microarrays can carry the specific antibodies that can recognize the characteristic proteins on the surface of exosomes. In combination with dip-pen nanolithography, highly selective capture of extracellular vesicles (EVs) is allowed. Liu et al. (2021) used lipid dip-pen nanolithography (L-DPN) to form the micron or nanoscale patterns of lipid patches on various substrates. Lipid membranes of DOPC (Fox et al., 2021) with a 5 mol% admixing of the biotinylated phospholipid cap biotinyl are obtained *via* L-DPN, which is then incubated with streptavidin solution to provide the binding sites for biotinylated antibodies. The target EVs can be captured as EVs pass through the array. EV cargos are eventually captured in the membrane patch through the membrane fusion (Figure 5). Highly sensitive recovery with only a small volume of liquid can be achieved by lipid microarrays. At the same time, the microarray has inherent anti-fouling properties, providing a basis for downstream genomic and proteomic analysis. To visualize EVs, EVs can be clearly observed under the fluorescence microscopy by using the lipophilic dye staining (e.g., PKH26). The lipid microarray platform can effectively capture EVs from MCF7 cell conditioned medium verified by various controls/experiments. Besides, functional EVs can be screened by the anion-exchange method (Seo et al., 2022). However, this microarray is suitable for large scale isolation of biologically active exosomes.

#### 3.2.2 Capture by immunomagnetic beads

**FIGURE 6:**
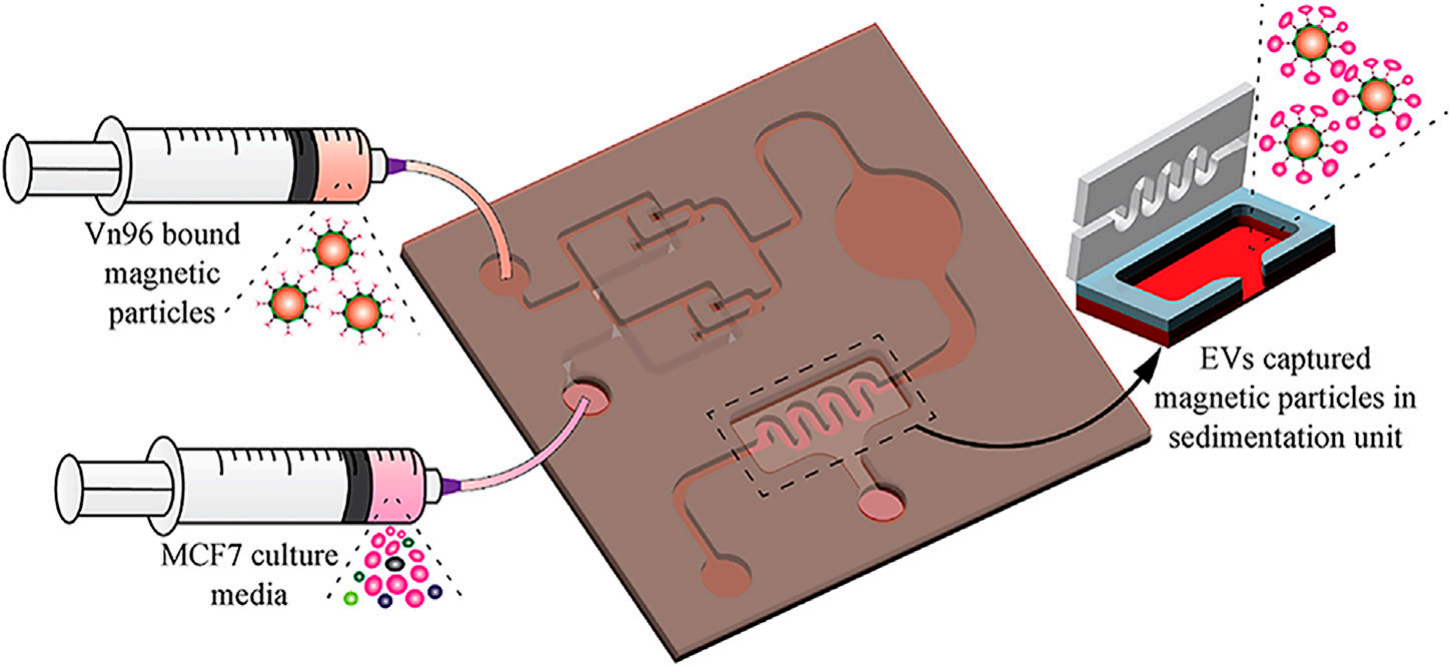
Scheme of the EV-isolation from MCF7 CCM. Vn96-bound magnetic particles are injected into the liquid biopsy chip simultaneously with MCF7 CCM at the same flow rate for EVs isolation. EVs are captured by the magnetic particles in the sedimentation unit. Reproduced with permission from (Bathini et al., 2021).

Liquid biopsy chips are gradually becoming the mainstream for the integration of the isolation and identification of exosomes. Bathini S et al. proposed a magnetic particle-based chip to isolate EVs by using the synthetic peptide Vn96 (Bathini et al., 2021). Vn96 can bind to the heat shock protein (HSP) expressed on the surface of EVs (Taylor et al., 2020; Roy et al., 2021). A 3D mixer and a sedimentation unit are included in the chip. The streptavidin-coated magnetic particles were co-incubated with Biotin-PEG-Vn96 in a centrifuge tube for 30 min to become the Vn96-bound magnetic particles, followed by collected into syringes. Equal amounts of Vn96-bound magnetic particles were injected into the liquid biopsy chip simultaneously with MCF7 CCM at the same flow rate. The particles fully bound within the chamber start settling within 0.5 mm from the beginning of the chamber. The precipitate is finally collected (Figure 6). Then EVs are eluted and characterized, combined with the droplet digital PCR for quantification of the housekeeping gene (Pan et al., 2020). The bioactivity and morphological structure of EVs can be maintained by the chip. Similar to lipid microarray, it can be used for early diagnosis of diseases. In addition, Park et al. (2021) proposed a high-throughput integrated magneto-electrochemical device (HiMEX) which is a 96-well assay that can be used for clinical high-throughput EVs analysis. This assay was able to enrich EVs by antibody-coated magnetic beads and quantitate the proteins bound to EVs by electrochemical detection after enzyme amplification.

**FIGURE 7:**
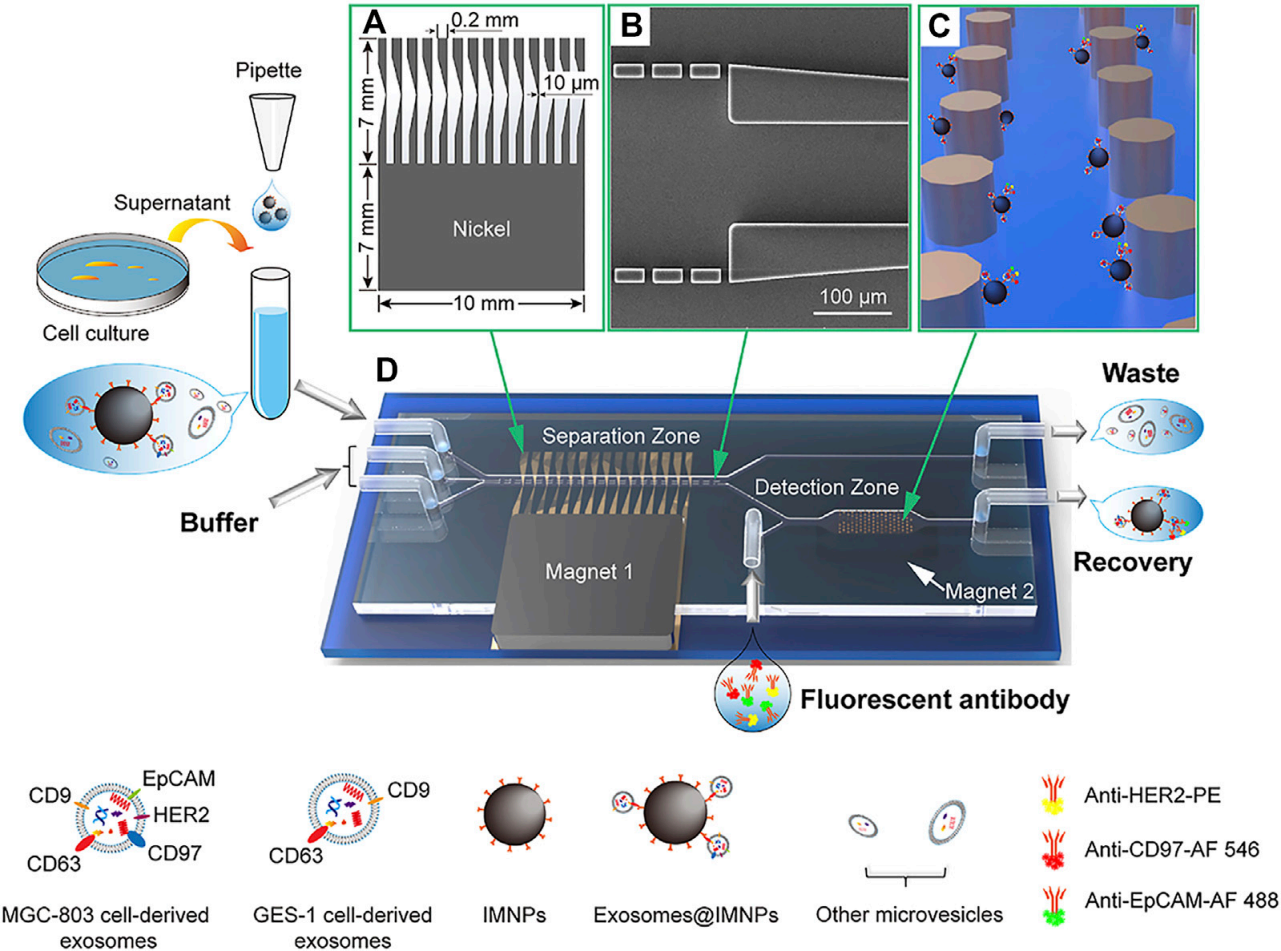
ExoSD chip for exosome separation by IMNPs. The separated exosomes@IMNPs were captured on the Ni cylinder array and labeled with fluorescent antibodies for detection. **(A)** Scheme of the Ni comb-like structure for local magnetic field and magnetic field gradient enhancement. **(B)** SEM image of the microfilter. **(C)** Schematic diagram of capturing exosomes@IMNPs by Ni cylindrical array. **(D)** Three-dimensional diagram of the ExoSD chip. Reproduced with permission from (Yu et al., 2021).

The highly integrated Exosome Separation and Detection (ExoSD) chip proposed by Yu et al. (2021) is used to extract exosomes from cell culture supernatants in a continuous flow manner, followed by the detection of exosomes by immunofluorescence. ExoSD chip contains a separation zone and a detection zone. Exosomes in the cell culture supernatant were captured by immunomagnetic nanoparticles (IMNPs). The mixture was injected into the ExoSD chip. The Exosomes@IMNPs will be separated in the separation zone from the mixture. The separated Exosomes@IMNPs were flowed into the detection zone and were captured on the Ni cylinder array. The nickel (Ni) comb-like structure was used to enhance the magnetic force on IMNPs. Finally, the cancer cell-derived exosomes will be labeled with fluorescent antibodies (Figure 7). ExoSD chip has gradually become a multifunctional platform for exosome isolation and detection. The recovery of exosomes was more than 80%, and the purity was more than 83% at the injection frequency of 4.8 mL/h. Exosomes can be detected by the plasma-coupled electrochemiluminescence immunosensors (Xiong et al., 2022).

Cheng et al. (2022) proposed a system for the isolation and release of exosomes, called immunomagnetic hedgehog particles (IMHPs). TiO_2_ bundles were assembled into hedgehog-type TiO_2_ particles (TiO_2_HPs) with specific spikes by using a hydrothermal method. Then magnetic responsive nanoparticles Fe_3_O_4_ with redox responsive disulfide bonds and CD63 antibodies were immobilized on the TiO_2_HPs to obtain TiO_2_@Fe_3_O_4_-anti-CD63HPs (IMHPs). The capture efficiency of exosomes isolated from MCF-7 cells by IMHPs was up to 91.70%. Exosomes were released by reducing the disulfide bonds on IMHPs by Tris(2-carboxyethyl)phosphine (TCEP) with the release efficiency of up to 82.45%. In addition to high isolation efficiency, the bioactivity and structural integrity of exosomes can be maintained by IMHPs compared with traditional isolation methods. To reduce the pretreatment steps for the exosome isolation, Boriachek K et al. used CD63 antibodies functionalized gold-loaded iron oxide nanocubes (Au-NPFe_2_O_3_NC) to directly isolate exosomes (Boriachek et al., 2019).

**FIGURE 8:**
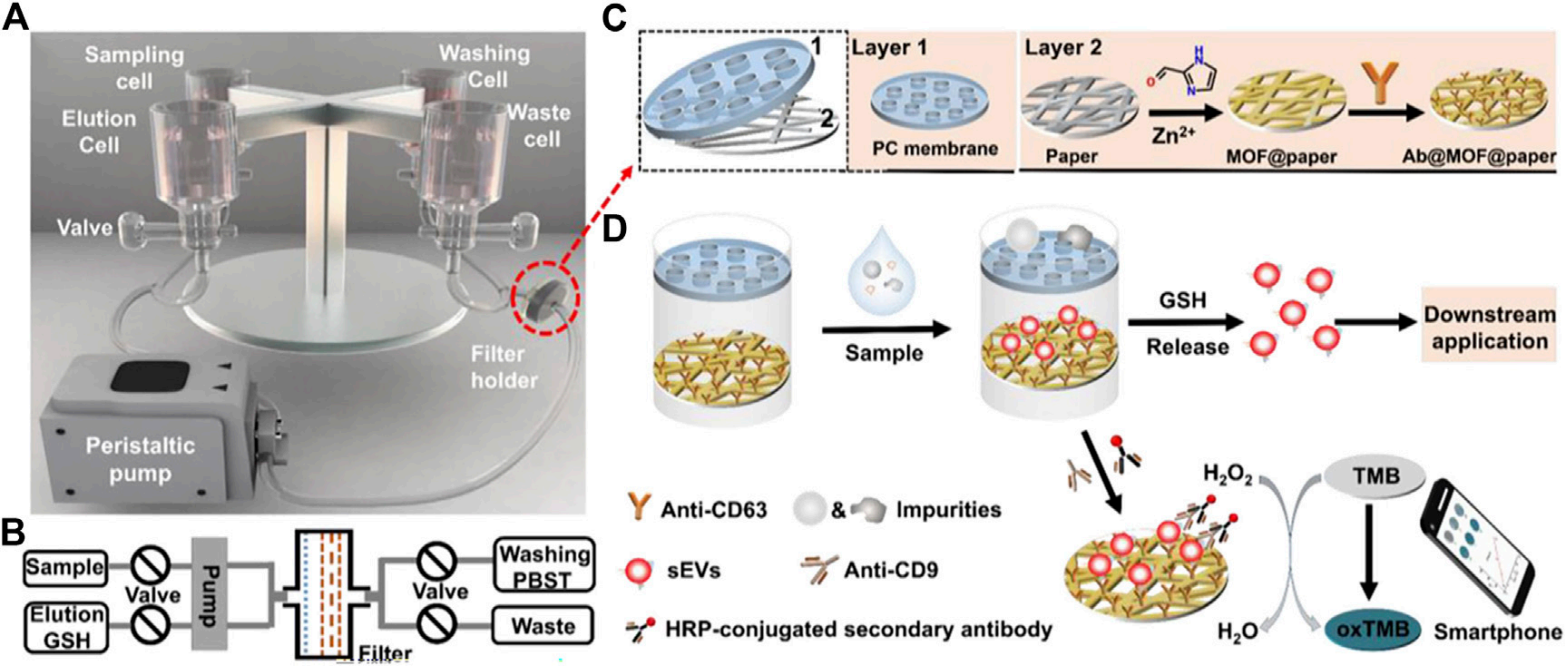
Schematic of the sEV-IsoPD. **(A)** The device consists of a peristaltic pump, four-cell container, valves, and filter holder. **(B)** Schematic diagram of the flow system of sEV-IsoPD. **(C)** Scheme of the PC membrane and the fabrication of Ab@MOF@paper. **(D)** The process of capturing, releasing, and detecting sEVs by sEV-IsoPD. Reproduced with permission from (Seo et al., 2022).

3D Paper-Based sEV isolation system (sEV-IsoPD) is used for the rapid isolation, and the advantages of high yield and purity can be provided (Zhang et al., 2022c). sEV-IsoPD consists of a peristaltic pump, a filter holder, and four cell containers. The filter holder is composed of a polycarbonate porous membrane for size exclusion and metal-organic framework (MOF)/CD63 antibody-modified paper (Ab@MOF@paper). EVs can be isolated from serum and cell culture medium by Ab@MOF@paper. After being captured by Ab@MOF@paper, sEVs can be released by catabolizing glutathione from ZIF-90 (Figure 8). Compared to ultracentrifugation and filtration, higher yield exosomes can be recovered in a short time. In addition to this, microfluidic chips consist of polydimethylsiloxane and glass to specifically capture EVs by using CD63 (Song et al., 2020) and PTK7 aptamers in the microchannel. CLIKKPF (the higher affinity peptide) was immobilized on SiO_2_ microspheres to capture exosomes by the high affinity between phosphatidylserine and CLIKKPF (Yang et al., 2022b). Kim et al. (2022b) pointed out that EVs can be captured by adsorption of antibodies (e.g., CD63 antibody) onto a citrate-covered plasma gold surface by physical adsorption, which showed higher efficiency and specificity.

Zheng et al. (2022) designed a microfluidic chip with a teardrop-shaped micropillar array. Exosomes were captured by Tim4-modified magnetic beads (Tim4 beads). The teardrop-shaped microcolumn arrays play an important role in the trapping process. The capture of exosomes by Tim4 beads is dependent on Ca^2+^. The intact exosomes can be released by using the chelators. Therefore, the bioactivity and morphological integrity of exosomes can be maintained with high efficiency and sensitivity. Moreover, the DNAzyme-triggered system was proposed to aggregate individual sEVs into clusters (Yu et al., 2022a). In this system, DNAzyme probes with cholesterol tails was hybridized to the CD63 aptamer-modified substrate probes to enrich exosomes. Exosomes can be rapidly isolated without ultracentrifugation. The reproducible temperature-sensitive electrochemical biosensor was constructed to detect exosomes (Liu et al., 2022a).

### 3.3 Capturing exosomes with covalent chemistry

The specific purification of Ewing sarcoma (ES)-derived EVs can be achieved by using the ES-EV Click Chip (Dong et al., 2020). Si nanowires are embedded in the microchip, and then combined with covalent chemistry-mediated capture of EVs. The chip consists of a Tz-grafted SiNWS and a PDMS-based chaotic mixer with a serpentine microchannel. Tz was immobilized on Si nanowires by the reaction of the Tz-sulfo-NHS ester with the NHS ester on the SiNWS, yielding the Tz-grafted SiNWS. The chaotic mixer is combined with Tz-grafted SiNWS by microfluidic scaffolding to make a complete ES-EV Click chip. LINGO-1 is a specific marker of ES which was found to be expressed on the surface of ES EVs by immunogold-TEM. ES EVs can be specifically recognized and conjugated by anti-LINGO-1. The primary amine group on anti-LINGO-1 was reacted with TCO-PEG4-NHS ester to form TCO-anti-LINGO-1 conjugate. The inverse electron demand Diels–Alder (IEDDA) reaction between the Tz and TCO moieties was used to capture EVs on the Tz-grafted SiNWS. EVs were released *via* the disulfide bond cleavage agents (such as 1,4-dithiothreitol, DTT). Specific purification of ES-derived EVs is allowed by EVs ES-EV Click Chip, while the integrity of EVs is maintained. The chip is suitable for the study of downstream functions of ES EVs.

**FIGURE 9:**
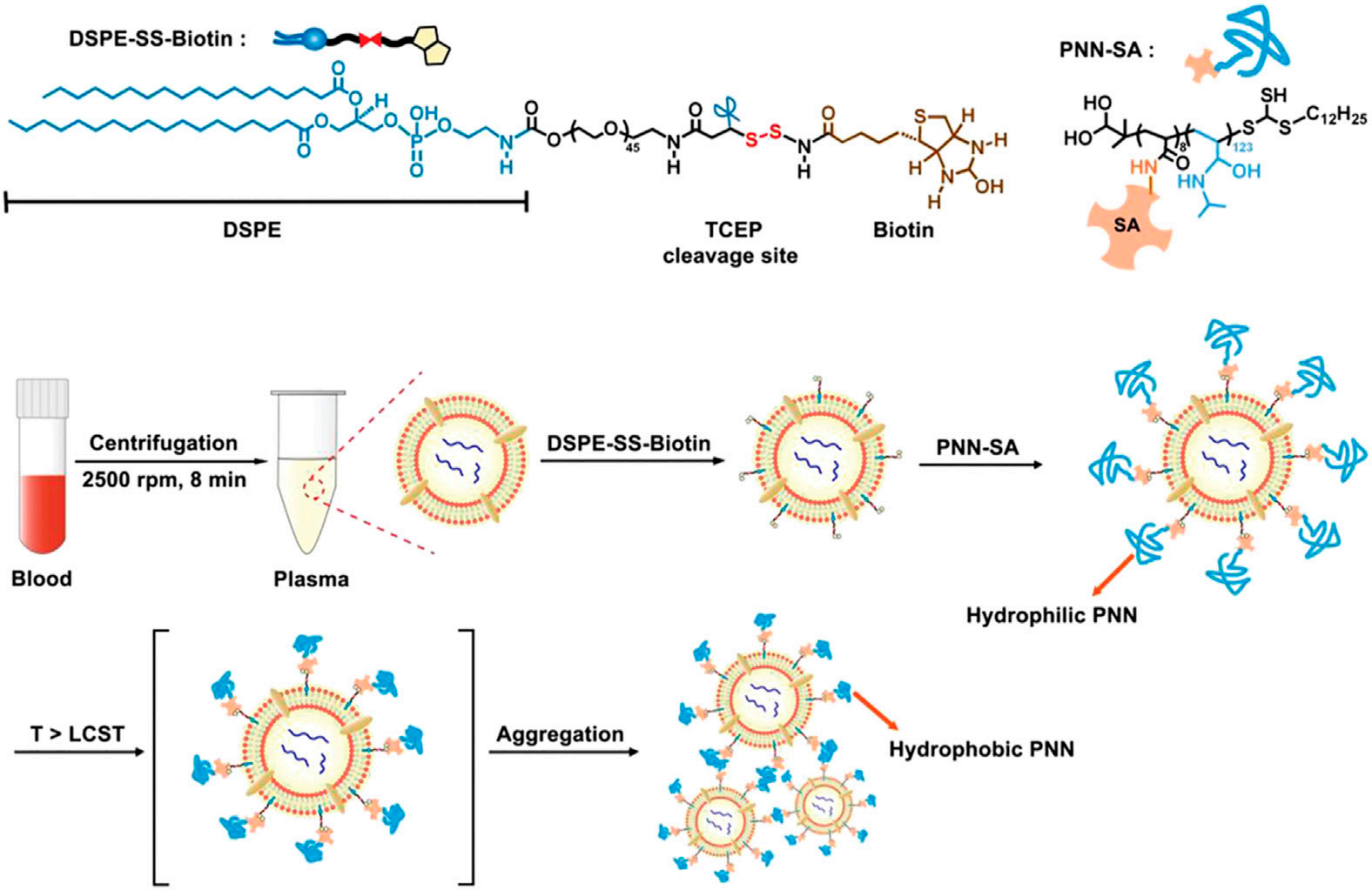
The molecular structures of PNN-SA and DSPE-SS-Biotin. DSPE-SS-Biotin can be inserted into the lipid bilayer of exosomes, and bare biotin interacts with PNN-SA to capture exosomes. The process is temperature-dependent. Reproduced with permission from (Zhang et al., 2022c).

Liu X et al. proposed a stimulus-mediated exosome enrichment and purification system for efficient extraction of exosomes from plasma samples (Liu et al., 2022b). Targeted enrichment and purification of exosomes can be achieved by mounting specific stimulus corresponding copolymers on lipid bilayers of exosomes. N-isopropylacrylamide (NIPAM) and N-acryloyloxysuccinimide (NAS) were polymerized *via* reversible addition-fragmentation chain transfer (RAFT) to form PNN. PNN-SA was formed by PNN and streptavidin (SA) *via* a carbodiimide cross-linker (Figure 9). DSPE-SS-Biotin inserted into the lipid bilayer of exosomes, with the biotin tail exposed. PNN-covered exosomes (PNN-Exos) was produced by the interaction between the anchored biotin tag and PNN-SA. The lower critical solution temperature (LCST) is 31°C. PNN dissolves in aqueous solution at room temperature 25°C (T < LCST). With the increasing temperature (T > LCST), PNN undergoes reversible hydrophilic-hydrophobic collapse, leading to phase separation and precipitation, and the solution changes from clear to turbid. Compared with traditional isolation methods (e.g., ultracentrifugation, ExoQuick), the bioactivity of exosomes can be effectively maintained by this system with higher yield and purity. In addition, the system can be combined with specific analytical tools to characterize relevant exosome biomarkers such as microRNAs. However, this system cannot be applied to larger EVs, and the validation in larger clinical cohorts for prospective studies is required.

In order to achieve rapid capture of EVs, Sun et al. (2022) proposed the concept of Click Beads. TCO-PEG-NHS was co-incubated with DSPE-PEG_1000_-NH_2_ to form DSPE-PEG_1000_-TCO conjugates. EVs were labeled by inserting the lipid motif of DSPE-PEG_1000_-TCO into the EVs membrane. TCO-labeled EVs were captured on Click Beads by a bioorthogonal click chemistry reaction between TCO and Tz motifs. Isolated exosomes on Click Beads were collected in tubes by the centrifugation. Compared to traditional isolation methods, Click Beads are time-saving and efficient, allowing for the capture of multiple cancer-derived exosomes and downstream analysis by RT-dPCR. Moreover, further analysis of exosomes can be attempted in combination with the one-step thermophoretic AND gate operation (Tango) assay (Deng et al., 2022).

**FIGURE 10:**
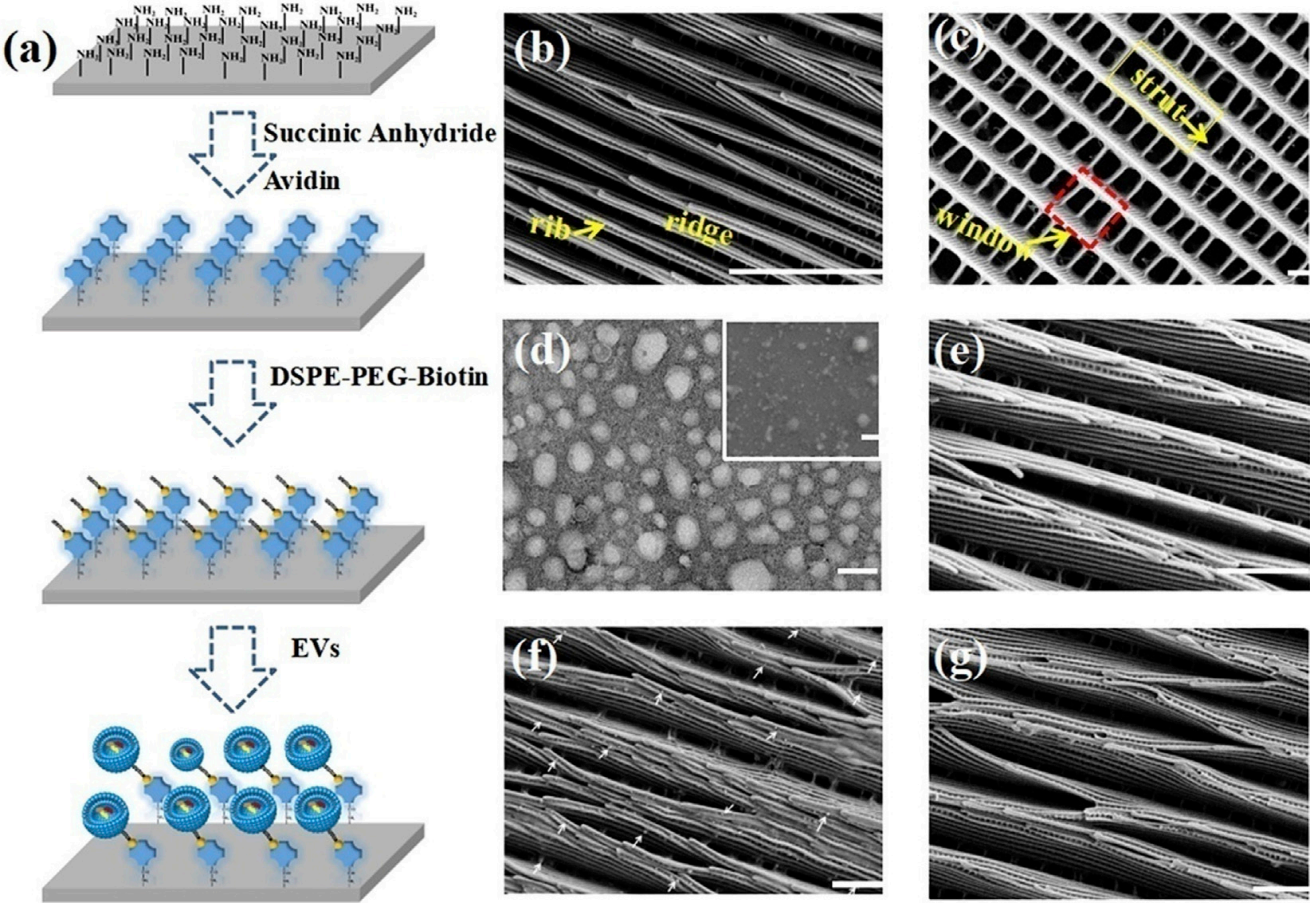
Modification of M. Menelaus wing by lipid nanoprobes and SEM images of M. Menelaus wings at different stages of the process. **(A)** Schematic diagram of the modification process of lipid nanoprobes on the surface of M. Menelaus wing. **(B, C)** SEM image of wing structure. **(D)** TEM image of the EVs. **(E)** SEM image of EVs captured by M. Menelaus wing without lipid nanoprobes modification. **(F)** SEM image of EVs captured by lipid nanoprobes modified M. Menelaus wing (as shown by the white arrow). **(G)** SEM image of EVs captured by M. Menelaus wing after treatment with triethylamine. Reproduced with permission from (Han et al., 2020).

It is difficult to efficiently isolate exosomes from biofluids, in order to solve the problem, a new microvortex chip has been constructed. EVs can be efficiently separated by integrating lipid nanoprobe modified Morpho Menelaus (M. Menelaus) wings into microfluidic chips (Han et al., 2020). DSPE-PEG-biotin lipid nanoprobe solution was co-incubated with affin-coated M. Menelaus wings, and biotin-avidin reaction was used to obtain lipid nanoprobe modified M. Menelaus wings. The lipid tail can be inserted into the EVs membrane for the exosome capture. Also, the downstream analysis is allowed by the chip (Figure 10). GPC-1 mRNA from breast cancer cell-derived EVs is used for the functional validation. This novel microvortex chip was reported to achieve the high throughput enrichment of EVs with an efficiency of up to 70%. The natural M. Menelaus wing has a series of intersecting point-connected 3D microgroove structures arranged in parallel on the surface, generating microvortexes for EVs isolation.

### 3.4 Other strategies

Tulkens et al. (2020) combined ultrafiltration, size-exclusion chromatography, and density gradient centrifugation in sequence to isolate bacterial-derived EVs, ensuring the integrity of EVs and eliminating the need for labeling and facilitating the subsequent characterization of isolated EVs. In addition, exosomes can also be isolated by CD9-HPLC-IAC (Zhu et al., 2021) and untouched isolation (Yu et al., 2022b). Recombinant EVs are gradually developed for disease diagnosis and treatment (Geeurickx et al., 2019). The process of exosome uptake and content transport is becoming clear (Bonsergent et al., 2021). To solve the problem of natural exosomes being cleared from blood circulation in three hours, Lathwal et al. (2021) designed exosome-polymer hybrid to make exosomes more stable *in vitro*. Extracellular vesicle mimetics prepared by top-down or bottom-up strategies have great potential for clinical applications (Liu et al., 2022c).These approaches shown above all focused attempts to better isolate exosomes.

## 4 Challenges and future outlook

Exosomes have attracted extensive attention over the past few decades due to their potential clinical applications. Nowadays, many methods and technologies have been developed for the separation of exosomes. In this section, we discuss the challenges and prospects of current exosome separation methods. Due to the heterogeneity of exosomes and the complexity of the matrix, exosome separation at high purity, yield, and recovery remains a challenge. The current methods for separating exosomes are mainly dependent on their size and surface-specific proteins. Size-based methods are label-free, but they suffer from low purity. Immunoaffinity methods can isolate exosomes within a short time, but they depend on the specificity of the markers on the surface of exosomes. However, the commonly used markers are not entirely specific to exosomes. The selection of isolation method is very important for the follow-up research, for example, related studies on exosome genomics and proteomics, as well as the role of exosomes in the treatment of diseases.

### 4.1 How to improve the purity and recovery of exosomes

The low purity of exosome separation is mainly caused by the large number of co-isolated contaminants. In particular, co-isolated non-exosomal functional vesicles will affect the subsequent experiments. There are also a large number of co-isolated proteins that overlap characteristics with exosomes in terms of density and size. Therefore, it is difficult to isolate exosomes from biological fluids without contaminations, which may affect the purity of the isolated exosomes.

In order to improve the purity of isolation, the first problem to be solved is to identify the specific differences in size, density, surface protein, and other physicochemical properties between exosomes and other non-vesicle components. For example, immunoaffinity capture can obtain higher purity by selecting appropriate antibodies or aptamers to bind exosomes according to the surface-specific markers on exosomes. Currently, combinations of multiple methods are being applied for the isolation and recovery of high-purity exosomes, such as ultracentrifugation combined with size-exclusion chromatography. The comprehensive method has the excellent performance to improve the label-free recovery of exosomes with high purity. However, there are few studies on the isolation of exosomes by comprehensive methods. A lot of work needs to be done to promote the development of this method.

### 4.2 Bioactivity and morphological integrity of exosomes

Due to changes in external forces or microenvironment, some existing separation methods inevitably destroy the bioactivity and morphological integrity of exosomes. For example, high rotating external force and extrusion of filtration membrane will cause mechanical damage to exosomes. The bioactivity and structural integrity of exosomes can be effectively maintained by avoiding external damage reasonably, such as the use of size-exclusion chromatography which depends on natural gravity-based isolation. In addition, the bioactivity and integrity of exosomes may be affected by the physicochemical properties in different biological fluids or by substances that bind to exosomes specifically. Capture by covalent chemistry can also effectively preserve the bioactivity and integrity of exosomes, or by using easily isolated substances such as Ca^2+^-dependent Tim4 proteins. It is crucial to efficiently recover intact exosomes for the disease treatment and biology studies of exosomes.

### 4.3 High throughput and automated microfluidic

The microfluidic technology is considered to be highly promising because of low consumption, automation, and portability. However, the technology is high cost and low throughput. For example, due to inherent label-free features, the deterministic lateral displacement, dielectrophoretic, etc., show great potential for the recovery of exosomes. However, these methods suffer from relatively low purity. It is essential to improve the throughput and separation velocity. Although many microfluidic methods have been developed for exosome isolation, none are widely used in clinical practice. One of the main reasons is that there are limited samples available for clinical validation in microfluidic platforms. Some microfluidic chips can achieve good separation performance in culture medium. However, due to the complexity of the sample, a large number of clinical samples are needed to verify its reliability, sensitivity and specificity. However, clinical validation with a large number of samples is not easy and need us to make more efforts. We believe that the microfluidic device suitable for clinical exosome separation will be developed.

Despite the remarkable application prospects, there are still some problems worth discussing. The heterogeneity of exosomes is not well understood, which may derivatively influence the selection of different separation methods. With so many metrics to consider, such as purity, recovery, yield, etc., it is hard to say which method is the best option. At present, choice of the most appropriate methods must be based on each specific research requirements and application scenarios. The comprehensive method is also recommended by using multiple separation methods to improve purity and yield. It should be emphasized that the separation method chosen must be based on factors that may differ from studies. Therefore, there is no uniform method for exosome separation.

## 5 Conclusion

Appropriate isolation method/means is the premise of exosome research. Overall, more and more methods used for the isolation of exosomes have made great progress in maintaining the bioactivity and morphological integrity of exosomes. So far, many isolation methods can be used not only to guarantee high isolation purity, but also to ensure high isolation yield, which have a great functional improvement. However, each method has its own merits and demerits. The microfluidic systems also have the disadvantage of low sample capacity. The combination of methods can be used to solve certain problems through complementary advantages, but there are still limitations. Therefore, it is challenging to develop an integrated isolation device with the advantages of simplicity, convenience, high yield, high purity, and low cost. The road to standardization, integration, and high throughput of isolation equipment is obstacle-packed and long. The development of comprehensive microarrays is a promising method for exosome isolation, while the study of exosome subgroups and heterogeneity will be expanded in depth. It is believed that in the near future, researchers will make efforts on cutting-edge exosome isolation method for clinical application.
